# Acid stress response and protein induction in *Campylobacter jejuni* isolates with different acid tolerance

**DOI:** 10.1186/1471-2180-12-174

**Published:** 2012-08-13

**Authors:** Tina Birk, Monica Takamiya Wik, René Lametsch, Susanne Knøchel

**Affiliations:** 1 Department of Food Science, Faculty of Sciences Copenhagen University, Rolighedsvej 30, 1958, Frederiksberg C, Denmark; 2 Division of Food Microbiology, National Food Institute, Technical University of Denmark, Mørkhøj Bygade 19, 2860, Søborg, Denmark

## Abstract

**Background:**

During the transmission route from poultry to the human host, the major foodborne pathogen *C. jejuni* may experience many types of stresses, including low pH caused by different acids. However, not all strains are equally sensitive to the stresses. The aim of this study was to investigate the response to acid stress of three sequenced *C. jejuni* strains with different acid tolerances using HCl and acetic acid.

**Results:**

Two-dimensional gel electrophoresis was used for proteomic analysis and proteins were radioactively labelled with methionine to identify proteins only related to acid exposure. To allow added radioactive methionine to be incorporated into induced proteins, a modified chemically defined broth was developed with the minimal amount of methionine necessary for satisfactory growth of all strains. Protein spots were analyzed using image software and identification was done with MALDI-TOF-TOF. The most acid-sensitive isolate was *C. jejuni* 327, followed by NCTC 11168 and isolate 305 as the most tolerant. Overall, induction of five proteins was observed within the pI range investigated: 19 kDa periplasmic protein (p19), thioredoxin-disulfide (TrxB), a hypothetical protein Cj0706 (Cj0706), molybdenum cofactor biosynthesis protein (MogA), and bacterioferritin (Dps). Strain and acid type dependent differences in the level of response were observed. For strain NCTC 11168, the induced proteins and the regulator *fur* were analysed at the transcriptomic level using qRT-PCR. In this transcriptomic analysis, only up-regulation of *trxB* and *p19* was observed.

**Conclusions:**

A defined medium that supports the growth of a range of *Campylobacter* strains and suitable for proteomic analysis was developed. Mainly proteins normally involved in iron control and oxidative stress defence were induced during acid stress of *C. jejuni*. Both strain and acid type affected sensitivity and response.

## Background

*Campylobacter jejuni* is a leading cause of foodborne disease with poultry as a common vector. During the transmission route to the human host, *C. jejuni* may experience many types of stresses such as exposure to oxygen in the environment, large temperature shifts, and changes in pH. Compared with many other foodborne pathogens, *C. jejuni* is more sensitive towards stress such as acid [1-3] and has stringent requirements for optimal growth conditions [4].

During colonization of the human host, *C. jejuni* is exposed to low pH environments. At first, the bacteria are exposed to inorganic acid (H^+^) in the gastric fluid of the stomach and later to organic acids in the small intestine [5,6]. The capacity to counteract environmental stresses is fundamental for survival. Bacteria respond to decreases in pH by inducing different systems to maintain pH homeostasis. These systems may prevent entry of H^+^, extrusion of H^+^ from the cell, consumption of H^+^ in chemical reactions or the repair of damaged cellular material. In some bacteria, such as *Salmonella* and *Listeria,* exposure to acid can up-regulate the F_0_F_1_-ATPase [7,8] by hydrolysis of ATP pump H^+^ out of the cell [9]. Amino acid decarboxylation acid resistance systems are found in many bacteria [10-12], however, these systems have not been identified in *C. jejuni*[13].

Compared to other bacteria, *C. jejuni* is more sensitive to stress and has a limited number of stress regulators. *C. jejuni* lacks the global stationary-phase regulator, sigma factor RpoS, which induces expression of numerous proteins involved in different forms of stress responses [14] and it is also involved in acid stress in *E. coli* and *Salmonella*[15,16]. In addition, *C. jejuni* also lacks the oxidative stress response regulatory elements SoxRS and OxyR, and osmotic shock protectants such as BetAB [13,17]. However, *C. jejuni* does contain the global ferric uptake regulator (Fur) that regulates genes in response to iron transport, metabolism, and oxidative stress defence [18-20] and is involved in acid stress in *Salmonella* and *Helicobacter pylori*[21,22]. Compared with many other foodborne pathogens, *C. jejuni* is more sensitive to acid exposure [23]. This sensitivity is probably not only due to the lack of an acid resistance system but also to the lack of the mentioned regulatory proteins. How then does *C. jejuni* respond on the proteomic level when exposed to low pH?

Recently, a transcriptomic analysis of *C. jejuni* NCTC 11168 found changes in the expression of hundreds of genes upon acid shock or in a simulated gastric environment. Primarily, genes involved in encoding ribosomal proteins, transcription and translation, and amino acid biosynthesis were down-regulated [24]. Many of the genes up-regulated by acid stress in that study have previously been characterized as heat shock and oxidative stress genes [24]. However, microarray data are complex and all the up-regulated genes do not necessarily translate into changes in specific proteins vital for survival [25,26].

Here, we want to analyze the acid stress response of *C. jejuni* strains with different acid sensitivity. Since weak and strong acids have different modes of action on the bacterial cell [15,27], the acid induced response to both a weak acid, acetic acid, which can be encountered in foods) and a strong acid (HCl, which is found in the gastric fluid) was analyzed and compared.

Proteins synthesized during stress were labelled by incorporation of radioactive methionine and separated by two-dimensional (2D) electrophoresis. At first, a chemically defined broth (CDB) suitable for growth of different *C. jejuni* strains therefore had to be developed with minimal concentrations of methionine in order to minimize competition with radioactive methionine added upon stress exposure.

## Methods

### Bacterial strains and preparation of inocula

Three sequenced *C. jejuni* strains were tested for acid stress response: the clinical human isolate *C*. *jejuni* NCTC 11168 from the National Collection of Type Cultures, strain 305 (GeneBank accession number ADHL00000000 [28]) and strain 327 (GeneBank accession number ADHM00000000 [29]). Strains 305 and 327 were originally isolated from turkey production by Prof. Thomas Alter, Freie Universität, Berlin. Previous results (Birk et al. 2010, data not shown [23]) have found that strain 305 was less sensitive towards tartaric acid, and strain 327 was more sensitive to tartaric acid than the NCTC 11168, respectively. Strain 305 was denoted as acid-tolerant and strain 327 as acid-sensitive. During propagation and growth, all plates were incubated at 42°C in sealed gas jars under micro-aerobic conditions (5% O_2_, 10% CO_2_, 85% N_2_). Stocks of all strains were stored at −80°C in broth (BHI) (Oxoid CM225, England) containing 15% glycerol. From −80°C stocks, cultures were transferred to Blood Agar Base No. 2 (Oxoid CM271, England) supplemented with 5% horse blood and incubated for 3–4 days. One loop full of each culture was subsequently streaked onto new Blood Agar Base No. 2 plates. After 24 hours of growth, cells were harvested with 2 ml phosphate-buffered saline (PBS) (Oxoid BR0014, England). The harvested cells were adjusted to OD_600_ = 0.1 which has previously shown to correspond to approx. 8 log_10_ CFU/ml and subsequently used as inoculum.

### Preparation of chemically defined broth

A chemically defined medium, originally developed for *N. gonorrhoeae*[30], was modified in order to have an optimal broth to support growth of *Campylobacter* on plates. From the original medium, glucose was removed because *Campylobacter* is unable to ferment or oxidize hexose carbohydrates [31], and different amino acids were added. The required amino acids were determined from the amino acid metabolic pathway maps listed for *C. jejuni* NCTC 11168 in the Kyoto Encyclopedia of Genes and Genomes (KEGG) [32] Pathway Database. If metabolic enzymes were lacking or if the amino acid biosynthetic pathway was complex, the specific amino acid was added to the modified chemically defined broth (CDB).

Modified CDB was prepared in double-strength stock batches (see Table 1) without methionine and cysteine, which were added later. The double-strength CDB was stored at −20°C. Prior to the experiments, double-strength CDB was thawed at 4°C and diluted to single strength with MilliQ water (Table 1). Methionine and freshly prepared cysteine were added and the pH was adjusted to 7.0. Finally, the CDB was sterilized by filtration (pore size: 0.2 μm).

**Table 1:** **Components of modified chemically defined broth (CDB) for*Campylobacter jejuni***

**Components**	**Stock solution (mg/ml)**	**Vol stock solution (ml) for 1 liter**	**Final conc (mmol/l) of 1xCDB**
**Buffer solution** (10X)		100.0	
K_2_HPO_4_	34.8		20.0
KH_2_PO_4_	27.2		20.0
**Salt solution** (20X)		50.0	
NaCl	116.0		100.00
K_2_SO_4_	20.0		5.74
MgCl_2_, 6 H_2_O	8.2		2.02
NH_4_Cl	4.4		4.11
EDTA	0.074		0.01
**Amino acid mix 1** (100X)		10.0	
L-Arginine HCl	15.0		0.71
L-serine	5.0		0.48
L-leucine	9.0		0.69
L-isoleucine	3.0		0.23
L-valine	6.0		0.51
L-proline	5.0		0.43
L-phenylalanine	5.0		0.30
L-alanine	10.0		1.12
L-histidine	5.0		0.32
L-threonine	5.0		0.42
L-lysine	5.0		0.30
L-glycine	2.5		0.33
L-trypthophan	8.0		0.39
**Amino acid mix 2** (10X)		100.0	
L-aspartate	5.0		3.76
L-glutamate	13.0		8.83
**Individual amino acids**			
L-cysteine/HCl †	17.5	3.0	0.35
L-cysteine †	12.0	3.5	0.15
L-methionine	14.9	1.0	0.10
**Vitamin mix (50**X**)**		0.2	
NAD	10.0		0.0030
Thiamine HCl (Vitamine B_1_)	10.0		0.0060
Calcium pantothenate (Vitamine B_5_)	10.0		0.0040
**Individual components**			
Oxaloacetate, 2 H_2_O (10X)	2.5	100.0	
NaHCO_3_ (2000×)	84.0	0.5	1.49
Biotin (Vitamine H) (500X)	0.25	2.0	0.50
Fe(NO_3_)_3_, 9 H_2_O (1000X)	4.0	1.0	0.0021
CaCl_2_, 2 H_2_O (1000X)	37.0		0.01
			0.25
**Total (ml)**		372.2	
MilliQ water (ml) *		627.8	

The broth was prepared as described in the text.

*: To make portions of 500 ml 2 × CDB without methionine and cysteine, only 127.8 ml of MilliQ water was added. The batches were sterilized by filtration, aliquoted, and stored at −20°C.

†: Cysteine was prepared freshly and dissolved in 1 M HCl prior to each experiment.

Individual stock solutions were prepared before the mixing of CDB. 10X buffer solution and 20X salt solution were made, autoclaved for 15 min at 121°C, and stored at room temperature. The amino acid mix 1 (100X) was made in concentrations as listed in Table 1. However, methionine was prepared as an individual amino acid stock solution so the chemically defined broth could be prepared with different methionine concentrations. The amino acid mix, vitamin mix and the individual components were sterilized by filtration and stored at −20°C until use. Stock solutions of cysteine were prepared just prior to use.

### Growth in chemically defined broth

**Figure 1:**
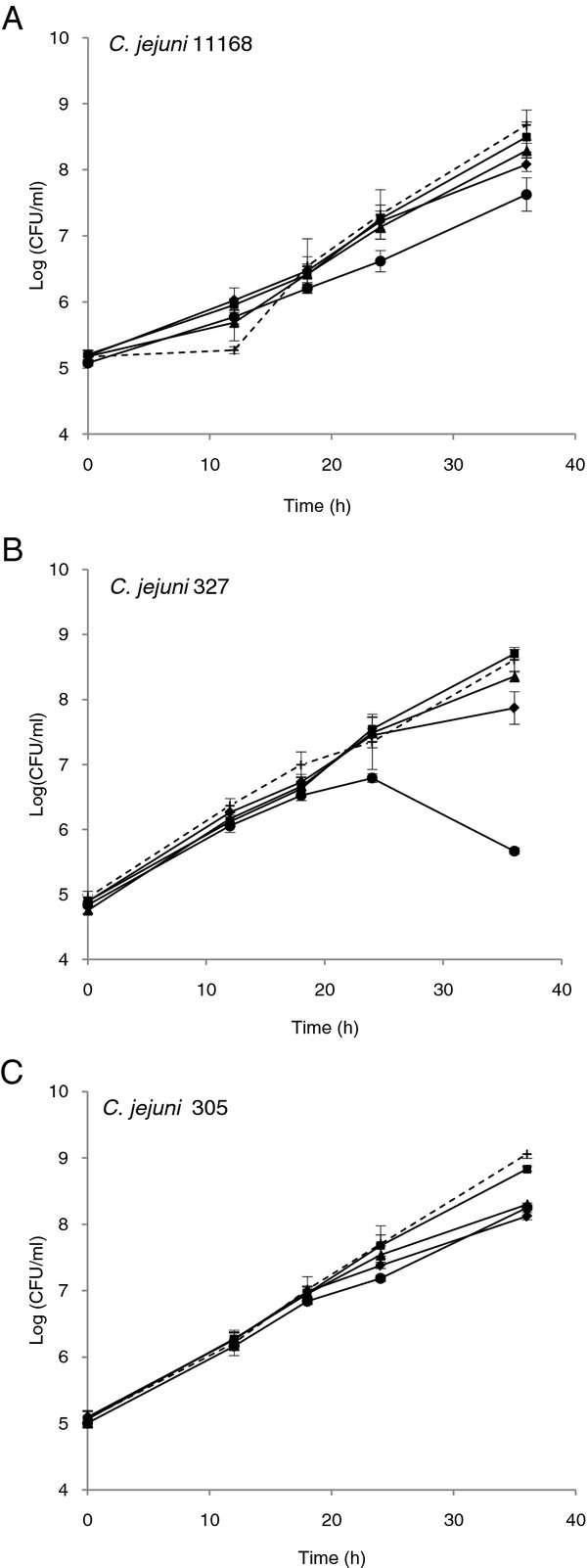
**Growth of the different *Campylobacter jejuni *strains in chemically defined broth (CDB) containing different concentrations of methionine.** Strains 11168 (A), 327 (B), and 305 (C) grown at 37°C in a microaerobic atmosphere in brain heart infusion (BHI) broth (dashed curve) and modified CDB containing 0.1 mM (■), 0.01 mM (▲), 0.001 mM (♦), and no (●) methionine, respectively. Error bars represent the standard deviation for three replicates.

In the growth experiment, *C. jejuni* strains NCTC 11168, 305, and 327 were tested for growth in CDB containing various concentrations of methionine (0.1 mM, 0.01 mM, 0.001 mM, and 0 mM) and compared with growth in BHI (Scharlau 02–1599, Spain) (Figure 1). From each inoculum, 12.5 μl was transferred to 25 ml pre-heated CDB (37°C) resulting in 4.95 (± S.D. = 0.21) log_10_ CFU/ml. Growth of another 10 strains was compared in BHI and CDB with 0.01 mM (data not shown).

### Microbiological analyses and sampling

*C. jejuni* cultures were serially 10-fold diluted in maximum recovery diluent (MRD) (Oxoid CM733, England) and 3 × 10 μl of appropriate dilutions were spotted onto Blood Agar Base No. 2 (Oxoid CM271, England) supplemented with 5% horse blood. After 24–48 h of incubation under microaerobic conditions, colonies were counted and the numbers of colony-forming units (CFU) per ml were determined.

### In vitro acid stress and [^35^ S]-methionine labelling and protein extraction

**Figure 2:**
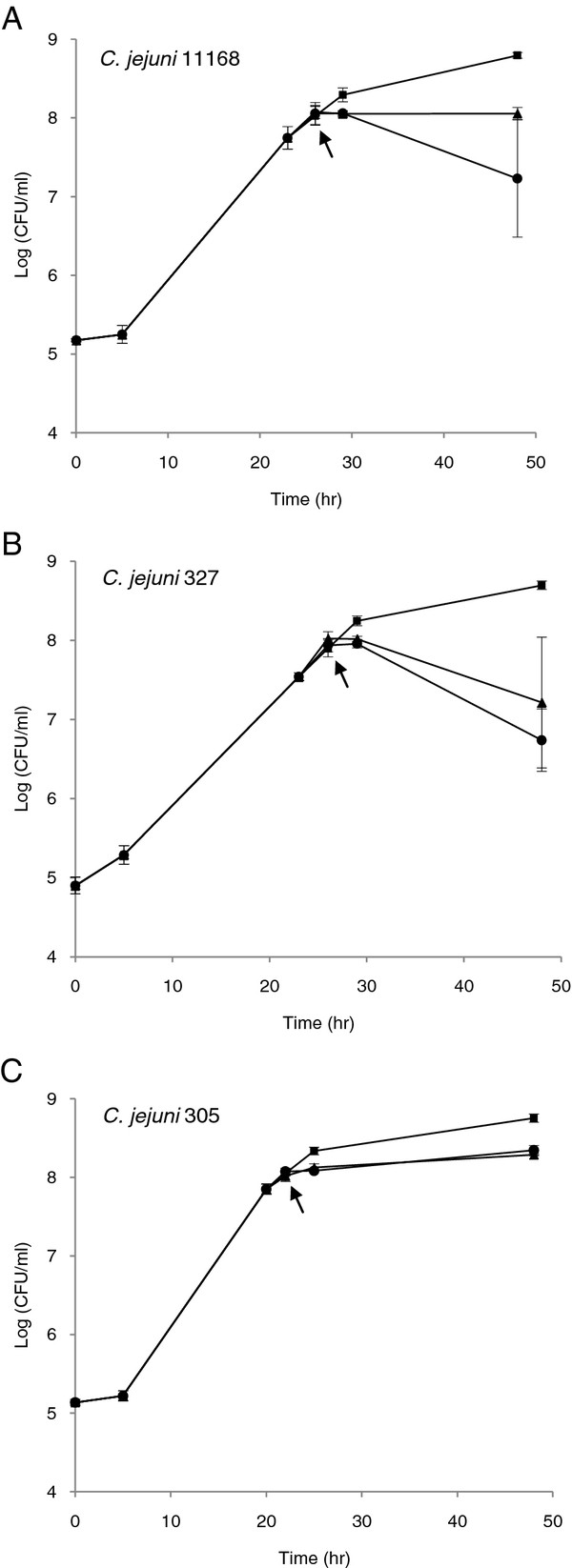
**Growth, acid stress and [**^**35**^**S]-L-methionine labelling.***C. jejuni *strains were grown to late exponential phase in modified chemically defined broth (CDB) containing 0.01 mM methionine at 37°C in a microaerophilic atmosphere. When cells had reached approximately 1 × 10^8^ CFU/ml, after 26 hours of growth for strains 11168 (A) and 327 (B) and after 22 hours for strain 305 (C), they were subjected to a shift in pH. The cells were first exposed to HCl (pH 5.2, ●) and acetic acid (pH 5.7, ▲) for 20 min before radioactive labelling with [^35^ S]-L-methionine for an additional 20 min. The control (■) was labelled for 20 min. The arrows indicate the point of labelling. After labelling, cells were harvested for proteome analysis. Data points are the mean of three replicates and standard variations are indicated by ± SEM (n = 3).

The response of *C. jejuni* to a strong acid (HCl) and a weak acid (acetic acid) was tested. These two different acids were selected because *Campylobacter* encounters HCl in the stomach and may be exposed to acetic acid during food processing. The cell cultures were adjusted to pH = 5.2 for HCl and pH = 5.7 for acetic acid since these values reduced growth rate to the same level for the most acid-tolerant strain 305 (Figure 2C). Three independent biological replicates were performed for each strain under two different acid stress conditions.

From the inoculum, 100 μl were transferred to 200 ml pre-heated CDB (37°C) containing 0.01 mM methionine resulting in approximately 5 log_10_ CFU/ml. *C. jejuni* strains NCTC 11168, 305, and 327 were grown to late exponential phase at 37°C to ensure high metabolic activity and overcome problems due to very low protein outcome in earlier phases (data not shown). After 26 hours of growth for strains 327 and NCTC 11168 and 22 hours for strain 305, the number of cells corresponded to approximately 8 log_10_ CFU/ml. Then 50 ml of the cell cultures (start pH about 7.0) were adjusted to pH 5.2 with HCl and pH 5.7 with acetic acid. Immediately after 2 × 1 ml cells were transferred to two tubes with screw cap, incubated for 20 min and labelled with 77 μCi/ml L-^35^ S]-methionine (Perkin Elmer, NEG-709A EasyTag^TM^™) for an additional 20 min at 37°C. The 40 minutes exposure was chosen to reduce the effect of acid shock [33].

After acid exposure, the cells were decanted by centrifugation at 18,620 × *g* (Hermle Z233) for 3 min. For extraction of proteins, extraction buffer [7 M urea (GE-Healthcare 17–131901), 2 M thiourea (Sigma-Aldrich, T7875), 4% CHAPS (GE-Healthcare, 17-1314-01), IPG buffer 4–7 (GE-Healthcare, 17-6000-86), 20 mM dithiothreitol (Sigma-Aldrich D-9779), 30 μg/ml chymostatin (Sigma-Aldrich, C7268), 15 μg/ml pepstatin (Sigma-Aldrich, P4265), 174 μg/ml phenylmethylsulfonyl fluoride (Sigma-Aldrich, P7626)], and 50 mg glass beads (D = 1 mm, Struers Kebolab, 115-790-1) were added for cell lysis in a FastPrep at speed 6 for 45 seconds. The suspension was centrifuged at 4°C at 18,620 × *g* (Hermle Z233) for 10 min and exactly 2 × 30 μl of protein sample was transferred to a clean Eppendorf tube and prepared for 2D gel electrophoresis.

### Two-dimensional gel electrophoresis

The protein sample was analyzed by using the GE-Healthcare Multiphor II Electrophoresis Systems using Immobiline DryStrips for the first dimension and the Bio-Rad Criterion Cell system for the second dimension. For rehydration, 60 μl protein sample was mixed with 140 μl rehydration solution [7 M urea (GE-Healthcare 17–131901), 2 M thiourea (Sigma-Aldrich, T7875), 4% CHAPS (GE-Healthcare, 17-1314-01), IPG buffer 4–7 (GE-Healthcare, 17-6000-86), 20 mM dithiothreitol (Sigma-Aldrich D-9779), and a few grains of bromphenol blue (Merck, 1.59102)] and applied to an 11-cm Immobiline DryStrip pH 4–7 (GE Healthcare, 18-1016-60) and the electrofocusing was run for a total of 18.2 hours (step 1: 300 V, 1 MA, 5 W, 0.01 h; step 2: 300 V, 1 MA, 5 W, 8 h; step 3: 3500 V, 1 MA, 5 W, 5 h; and step 4: 3500 V, 1 MA, 5 W, 5.20 h). Before protein separation by their molecular weight, the Immobiline DryStrips were equilibrated, first in 20 ml equilibration buffer [6 M urea (GE-Healthcare 17–131901), 50 mM Tris–HCl (Trizma Base, Sigma T-1503, pH 6.8), 30 v/v% glycerol (Merck, 1.04094), 2 w/v% SDS (GE-Healthcare, 17-1313-01)] containing 0.625 w/v% dithiothreitol (DTT) (Sigma-Aldrich D-9779) for 15 min and then in 20 ml equilibration buffer also containing 2.5 w/v% iodoacetamide (Sigma-Aldrich, I6525) and a few grains of bromphenol blue (Merck, 1.59102) for 15 min. In the 2^nd^ dimension, the Criterion^TM^ precast 10%–20% Tris–HCl Gel (Bio-Rad, 345–0107) gel was used for separation of proteins by size. After draining, the strips were sealed and connected to the gel by using 0.5% agarose and run in Laemmli running buffer [(30.3 g/l Trizma base (Sigma-Aldrich, T6066), 144 g/l glycine (Merck, 1.04201) and 10.0 g/l SDS (GE- Healthcare, 17-1313-01)]. The gels were stained using a silver staining kit (GE-Healthcare, 17-1150-01), coated with cellophane, dried overnight at room temperature, and exposed to phosphorus screens for 72 h.

### Image and data analysis

Radioactive proteins were visualized using a PhosphorImager (STORM 840, GE-Healthcare), and the protein spots were analyzed using the Image Master^TM^ 2D Platinum (version 5.0, GE-Healthcare). Initially, protein spots of one set of gels were matched and specific proteins that had higher intensity values than proteins from the control gel were annotated. One set of gels included HCl and acetic acids stressed cells plus a control as a reference. For comparative protein analysis, corresponding protein spots for each specific protein on the control, HCl, and acetic acid gels were manually defined as one group and the match was automatically verified before estimating the volume intensity. The three replicates were compared by normalizing the estimated volume intensity for the individual proteins to percent volume intensity for each replicate. The percent volume intensity was calculated for the specific conditions (control, HCl and acetic acid) as follows:% volume intensity control condition _(protein x)_ = volume intensity condition/(volume intensity control + volume intensity HCl + volume intensity acetic acid).

### In-gel digestion of protein spots

To examine relevant protein spots, *C. jejuni* cells were exposed to acid stress without labelling them with methionine but the proteins were separated using the same procedure as for labelling. After the 2^nd^ dimension, and fixation in equilibration buffer [concentrated H_3_PO_4_ (VWR, 20621.295), 150 g/l ammoniumsulfate (Merck, 1.01217), 18% ethanol] for 30 min, the gel was stained with 1 ml 20.0 g/l Coomassie Brillant Blue 250 G (Merck, 1.15444). Relevant protein spots were excised from the gel. The gel pieces were then washed and digested with trypsin as described by Sørensen et al. (2009) [34].

### Desalting, concentration, and loading on MALDI target

Gel-loader tips (Eppendorf) packed with Poros reverse phase 20 R2 (Applied Biosystems, 1-1128-02) was used as chromatographic columns for desalting and up-concentration of the digested protein sample prior to spectrometric analysis. The peptide digest was treated and loaded on MALDI target as described by Sørensen et al. (2009) [34].

### Identification of proteins by MALDI-TOF MS

A MALDI-TOF-TOF instrument (4800 Proteomics analyzer, Applied Biosystems, Foster City, CA) was used to identify proteins. The MS/MS spectra were analysed using Data Explore v4.6 (Applied Biosystems). Mascot MS/MS Ions Search (Matrix Science, http://www.matrixscience.com) was used to search for matching protein sequences within the NCBI database ( http://www.ncbi.nlm.nih.gov/). The taxonomy was restricted to *C. jejuni.* The mass tolerance was limited to 70 ppm for peptide mass fingerprinting and to 0.6 Da for peptide sequence data.

### Primer design and quantitative real time PCR (qRT-PCR) validation of proteome data

To examine if there is any correlation between induced proteins during acid stress with changes in mRNA level, a qRT-PCR study on *C. jejuni* strain NCTC 11168 was performed. Besides the induced proteins, the expression of the ferric uptake regulator (*fur*) was also included since it has been shown that Fur regulates genes involved in iron transport, metabolisms and oxidative stress defence [18-20]. The following were selected as internal and reference genes: *lpxC* (encoding UDP-3-O-[3-hydroxymyristoyl] N-acetylglucosamine deacetylase) [24] and *rpoA* (encoding the α-subunit of the RNA polymerase) (Table 2). The Primer Express software version 2.0 (Applied Biosystems) was used to design primers. PCR primers (Table 2) were purchased from TAG Copenhagen (Copenhagen, Denmark).

**Table 2:** **Primers used in qRT- PCR of *Campylobacter jejuni *NCTC 11168**

**Gene**	**product**	**Forward primer (5′ → 3′)**	**Reverse primer (5′ → 3′)**
*dps*	Bacterioferritin	TTCATAATCTTGTTTGATCA	AAGAGTTTTACAACTTGGCG
*cj0706*	Unknown	GATGAAGAAATCAAAGATATAG	CAAGAACTTCCATTTCAGAT
*sodB*	Superoxide dismutase	TATCAAAACTTCAAATGGGG	TTTTCTAAAGATCCAAATTCT
*trxB*	Thioredoxin- disulfide reductase	CAATGTATGCGTTTTGGTT	CAAGAACTTCCATTTCAGAT
*ahpC*	Alkyl hydroperoxide reductase	GTACTTTATGCAGAAGCAGT	CTACCTAGTGGTAAATCATT
*mogA*	Molybdenum cofactor biosynthesis protein	ACTTTCTAAGCGCATAAGTTCTCC	TACAAGCGGAGGTACAGGTC
*p19*	19 kDa periplasmic protein	GATGATGGTCCTCACTATGG	CATTTTGGCGTGCCTGTGTA
*fur*	Ferric uptake regulator	ACTCATTACACACCCGAA	TCACCACAAACACCATAAAG
*lpxC*	UDP-3-O-[3-hydroxymyristoyl] N-acetylglucosamine deacetylase	ATGAGTGCGATTAATGCTTA	GGCTTTTTAATTACCATAAT
*rpoA*	DNA-directed RNA polymerase subunit alpha	GCACCATAGGATATGCTCCAACT	CCACGCATGCTATCAAATTCAT

After acid stress exposure (same procedure as for the proteomic study), 3 ml culture was mixed with 6 ml RNA-protect (Qiagen) to stabilize RNA and incubated at room temperature for 30 minutes. The mixture was centrifuged. For enzymatic lysis of the cells, the pellet was dissolved in 100 μl TE buffer (30 mM Tris-Cl, 1 mM EDTA, pH 8.0) containing 15 mg/ml lysozyme, and added to 10 μl proteinase K (Qiagen) and incubated for 10 minutes at room temperature. For RNA purification and isolation, the RNeasy Mini Kit (Qiagen, 74104) was used and the included procedure was followed. To eliminate genomic DNA from the isolated RNA, the RNase-Free DNase set was used (Qiagen). First, the samples were measured out to 0.1 μg RNA thereafter cDNA was synthesized using the TaqMan Reverse Transcription Reagens (N8080234, Applied system). Each sample was prepared in triplicate resulting in a volume of 20 μl containing 5 μl cDNA, 10 μl 2 × Power SYBR green PCR mix (Applied Biosystems) and final concentration of 0.9 pmol/μl of forward and reverse primer.

For amplification of PCR products and quantification of produced cDNA SYBR Green, the 7500 fast real-time PCR system (Applied Biosystems) was used. The thermocycling conditions were 55°C for 2 min (uracil-N-glycolyase activation), 95°C for 10 min (Taq activation and uracil-N-glycolyase de-activation) followed by 40 cycles of 95°C min for 15 sec and 60°C for 1 min.

To determine the changes in the relative gene transcription level presented as fold changes, a mathematical model for relative quantification of in RT-PCR was used [35]. The expression level of the specific target during acid stress was compared with the expression level of non-stressed cells (control). Three individual biological experiments were performed and data presented as an average.

### Statistical analysis

All data from the growth experiments, comprising three replicates, were log transformed and statistically analyzed by SAS statistical software version 9.1 (SAS Institute, Cary, USA). To test for statistically significant differences in growth with various concentrations of methionine in CDB and BHI, a PROC GLM procedure was used. Volume intensity% differences between the individual proteins were calculated by variance analysis (ANOVA) in Microsoft Excel (version 2007).

## Results

### Growth in modified chemically defined broth

A modified defined broth that supports the growth of all three *C. jejuni* strains at the same level as in a rich medium (BHI) was developed (Figure 1). Ingredients used in the modified CDB for *C. jejuni* strains are shown in Table 1.

The change of protein synthesis during acid exposure was determined by adding radioactively labelled methionine to the modified CDB during the stress period. In order to minimize the competition between the methionine and the radioactive methionine in the modified CDB, the minimal concentration of methionine in the modified CDB was determined for the three strains. Strain 327 had a special requirement for methionine which was illustrated by the fact that in its absence, the bacteria started to die already after 24 h. This strain does not possess all the enzymes involved in synthesis of cellular methionine ( [29]). The modified CDB with 0.01 mM methionine was used in 2D gel analysis because no significant difference in growth was observed between this concentration and the highest concentration (0.1 mM) investigated (*P*_305_ = 0.07, *P*_11168_ = 0.36, *P*_327_ = 0.52) (Figure 1). The CDB with methionine supported good growth of all 13 strains tested. For nine of the strains the growth and generation times were comparable with BHI, while four of the strains showed either significantly faster or slower growth (unpublished observations). It has been shown that auxotyping markers, except cystine and cysteine, are stable after three cycles of freezing and thawing [30], and it is therefore possible to minimize the workload by preparing batches of double strength stocks and storing these at −20°C.

### [^35^ S]-methionine labelling during acid stress

*C. jejuni* strains NCTC 11168, 327, and 305 were grown in CDB containing 0.01 mM methionine at 37°C in a microaerophilic atmosphere. Similar numbers of cells in late exponential phase were desirable for comparability between the strains. To achieve cells in the late exponential phase with approximately 1 × 10^8^ CFU/ml, strains of NCTC 11168 and 327 were grown for 26 hours, whereas strain 305 only required 22 hours.

The *C. jejuni* cells were exposed to relatively mild acid conditions (pH 5.2 with HCl and pH 5.7 with acetic acid) to prevent the cells from dying and closing down all metabolic activity. The gastric pH during a meal has been measured to be 3.9-5.5 [36] and the experimental pH is therefore within the upper range. The effects of acid exposure on CFU for all strains are illustrated in Figure 2. Strain 305 was the most acid-tolerant strain while strain 327 was the most acid-sensitive at 37°C. This correlated well with earlier findings showing that strain 305 was more tolerant than strain 327 towards tartaric acid at 4°C [23]. Growth of *C. jejuni* 305 was only slightly reduced during HCl and acetic acid stress (Figure 2C), whereas the number of cells for strain 327 decreased (Figure 2B).

### Proteomic analysis and identification of proteins

**Figure 3:**
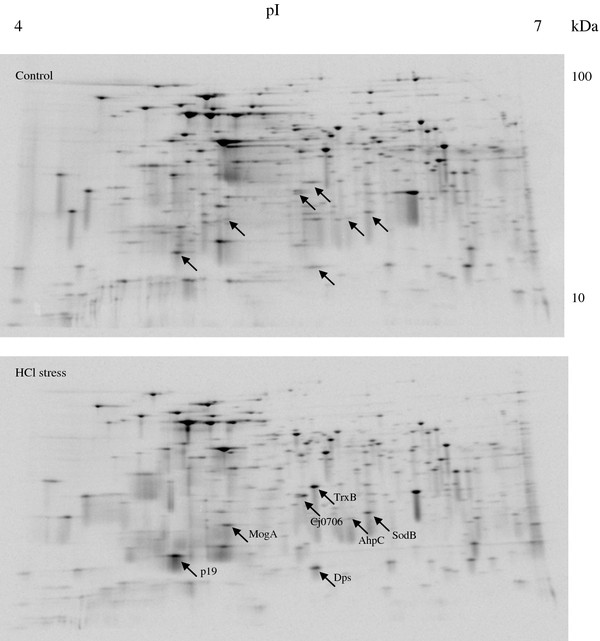
**Reference map of proteins from *C. jejuni *305 separated by 2D-gel-electrophoresis. **The strain was grown in modified chemically defined broth modified (CDB) containing 0.01 mM methionine at 37°C to late exponential phase and until the cell level was 1 × 10^8^ CFU/ml. Proteins were exposed to HCl (pH 5.2) for 20 min and then labelled with [^35^ S]-methionine for 20 min. Proteins were separated by their isoelectric point (pH 4–7) and then by their molecular weight on a 10%–20% Tris–HCl gel. The gel was scanned and only proteins, with incorporated [^35^ S]-methionine, were visible. Arrows point at induced proteins: 19 kDa periplasmic protein (p19), alkyl hydroperoxide reductase (AhpC), Superoxide dismutase (Fe) (SodB), Thioredoxin-disulfide reductase (TrxB), hypothetical protein (Cj0706), and molybdenum cofactor biosynthesis protein (MogA).

Methionine labelled protein extracts from non-stressed, HCl or acetic acid-exposed cells were subjected to 2D-gel-electrophoresis analysis. The majority of proteins were repressed as expected. Relatively few (up to seven) induced proteins were identified with only five being significantly induced. The intensity (% volume) was calculated for induced proteins under the following conditions: control, HCl, and acetic acid (Table 3). For strain NCTC 11168, four proteins [19 kDa periplasmic protein (p19), thioredoxin-disulfide (TrxB), hypothetical protein Cj0706 (Cj0706) and molybdenum cofactor biosynthesis protein (MogA)] were significantly induced (*P*_p19, HCl, Ac_ < 0.005, *P*_TrxB, HCl_ = 0.009, *P*_Cj0706, Ac_ = 0.016, *P*_MogA, HCl, Ac_ < 0.03). Volume% of bacterioferritin (Dps) during HCl stress was higher compared with the control, but probably due to the variation of the control this difference was not significant (*P*_11168, Dps, HCl_ = 0.061). For the acid-robust strain 305, Dps, p19, MogA and TrxB were significantly induced (*P*_Dps, HCl_ = 0.0028, *P*_p19, HCl_ = 0.0008, *P*_MogA, HCl_ = 0.018, *P*_TrxB, HCl_ = 0.017). Fewer proteins were induced in the acid-sensitive strain 327, which was also reduced during the acid stress (Figure 2B). Only induction of Cj0706 and MogA was observed during HCl acid stress (*P*_Cj0706, HCl_ = 0.0037, *P*_MogA, HCl_ = 0.04). In the case of NCTC 11168 and 305, the two proteins alkyl hydroperoxide reductase (AhpC) and superoxide dismutase (SodB) had higher% volume intensity than for the control indicating induction; however the differences were not significant. A reference profile of proteins separated by 2D-electrophoresis for *C. jejuni* 305 exposed to HCl stress (pH 5.2) is shown in Figure 3.

**Table 3:** **Induced proteins (% volume intensity) during HCl (pH 5.2) and acetic acid (pH 5.7) exposure in *C. jejuni *NCTC 11168, *C. jejuni *305 and *C. jejuni *327 at 37°C in chemically defined broth**

				***Campylobacter jejuni *strains**^**3**^		
**Protein/(NCBInr**^**1**^**)**	**Mw (kDa)**	**Score**^**2**^		**NCTC 11168**	**305**	**327**
Dps (NP282665)	17.4	222	Vol%			
p19 (CAA73983)	17.0	255	Vol%			
AhpC (NP281525)	22.0	668	Vol%			
SodB (NP281379)	25.0	241	Vol%			
TrxB (NP281357)	33.5	204	Vol%			
Cj0706 (NP281878)	28.0	431	Vol%			
MogA (YP_178829)	20.3	318	Vol%			
				C HCl Ac	C HCl Ac	C HCl Ac

Dps: Bacterioferritin, p19: 19 kDa periplasmic protein, AhpC: Alkyl hydroperoxide reductase, SodB: Superoxide dismutase (Fe), TrxB: Thioredoxin-disulfide reductase, Cj0706: hypothetical protein, MogA: Molybdenum cofactor biosynthesis protein.

Columns: light grey: control (C), dark grey: HCl stressed cells (HCl), white: Acetic acid stressed cells (Ac).

^1^ Identification was based on Mascot MS/MS Ion Search using sequence data from the database NCBInr.

^2^ Mowse Score (Score).

^3^ The intensity of the induced proteins was estimated by Image MasterTM 2D Platinum and % volume intensity was calculated.

The intensity of the protein spots was analyzed using the Image MasterTM 2D Platinum (version 5.0, Amersham Biosciences, Melanie). Three biological independent replicates was performed and % volume intensity was calculated as: % volume intensity control _(protein x)_ = volume intensity /(volume intensity control + volume intensity HCl + volume intensity acetic acid).

### Quantitative RT-PCR

**Figure 4:**
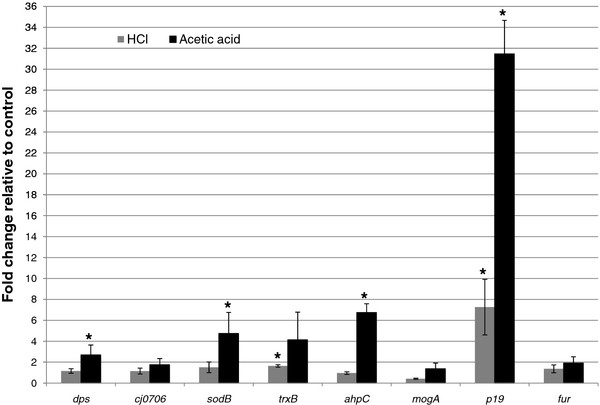
**Relative change in transcription level during acid stress of selected genes: *dps*, *cj0706*, *sodB*, *trxB*, *ahpC*, *mogA*, *p19 *and *fur *analyzed by qRT-PCR. *C. jejuni *strain NCTC 11168 was grown to 1 × 10**^**8**^**CFU/ml and exposed to HCl (pH 5.2) and acetic acid (pH 5.7).** The expression level of acid stressed for a specific gene was compared with unstressed cells and the horizontal line illustrates the fold change at 1.0 for the reference genes (*rpoA* and *lpxC*). Fold changes and standard deviations were calculated from the outcome of qRT-PCR runs from three microbiological independent experiments. Genes marked with an asterisk are significantly over-expressed compared with genes from non-stressed cells.

Transcriptomic analysis using qRT-PCR technique was performed to determine if the proteins induced during acid stress were induced at transcription level. Figure 4 illustrates the transcription profiles represented by fold change relative to control of *dps*, *cj0706*, *sodB*, *trxB*, *ahpC*, *mogA*, *p19* and *fur* during HCl and acetic acid stress for strain NCTC 11168. Interestingly, the transcriptomic data did not correspond completely with the proteomic data (Figure 4). The increased gene expression of *trxB* (*P*_HCl_ = 0.009) and *p19* (*P*_HCl, Ac_ < 0.05) during acid stress corresponded well with enhanced protein production. Especially noteworthy is the high acid stress response of *p19* gene compared with the other genes. Proteins such as SodB and AhpC, which were not significantly induced in NCTC 11168, were, however, over-expressed at transcription level during acetic acid exposure (*P*_*sodB*, Ac_ = 0.03, *P*_*ahpC*, Ac_ = 0.000). The regulator Fur was included in the qRT-PCR study because a search of putative Fur-regulated genes indicated that genes involved in iron-transport genes such as *p19*, *cj0178*, *ceuB*, *cfrA*, *chuA*, *exbB*, *feoB* and *cfhuA* and the iron-storage genes such as *dps*, ferritin (*cft*) and *cj0241* all contained Fur box promoters [37]. Fur was not induced in the proteomic study, but there was a tendency, however not significant, that *fur* was over-expressed during acetic acid stress (*P*_*fur*, Ac_ = 0.06).

## Discussion

Proteome analysis for *Campylobacter* during acid stress revealed different protein profiles between the strains and the type of acid used. The production of induced proteins during acid stress was measured using radioactive methionine labelling and 2D-gel-electrophoresis in a defined medium after the cultures had been exposed to a strong (HCl, pH 5.2) and a weak (acetic acid, pH 5.7) acid. Relatively few proteins (up to seven) were induced. However, only two were observed in the most acid-sensitive strain (327). The low number of induced proteins in this strain may be due to a shutdown of the metabolic activity as a result of cell death. In the sequenced strain NCTC 11168, both HCl and acetic acid exposure caused induction of proteins while in the most robust strain (305), marked protein induction was primarily seen with HCl. These differences reflect the strain variations in acid sensitivity and probably also the different mode of action of the strong and weak acid on the bacteria cell.

In a comparable proteomic study of the more acid-tolerant bacteria *E. coli* and *Salmonella,* a 1.5-4 fold induction of 13 proteins (*E. coli*) and a 2–14 fold induction of 19 proteins (*Salmonella*) were found when cells were shifted from pH 7 to 5 (phosphoric acid) [38]. The higher number of induced proteins in *E. coli* and *Salmonella* compared with what we observed may be due to the fact that *C. jejuni* lack the common acid resistance systems [10-12] and the global stress regulator protein RpoS, as well as the fact that the *C. jejuni* genome is small (1,660 kbp) [13]. Of course, small experimental differences and types of acid stress may influence the outcome as well.

The effect of the low pH on the bacterial cell is complex because it is interconnected with other factors such as oxygen stress, growth phase and produced metabolites [39]. Most of the proteins observed during acid stress in this study, such as SodB, AhpC, and Dps, have been associated with oxidative stress [40-43]. However, these proteins have also shown to be acid induced in *E. coli*[39,44,45], suggesting multiple protective mechanisms. This link has further been supported by a recent *Campylobacter* transcriptomic study where up-regulation of numerous genes including *ahpC*, *sodB* and *p19* during HCl exposure were reported [24]. The central role for Dps in acid tolerance response in *C. jejuni* is supported in a study with a *dps E. coli* mutant [45] and in an acid challenge study with *Salmonella*[26]. In *E. coli,* Dps has multi functional properties such as DNA binding, iron sequestration, ferrioxidase activity, and a central role for several stress responses – including acid stress [26]. Oxidative stress and free iron are closely connected [46], and it has been shown that decreasing pH results in enhanced iron-mediated lipid peroxidation processes [47]. Via the Fenton reaction, free iron can react with H_2_O_2_ and generate cell-damaging hydroxyl radicals (·OH) [48,49]. Regulation of free Fe^2+^ is therefore essential for cellular activities. Iron storage proteins indirectly contribute to oxidative stress defence by storing iron in an inactive form thereby preventing formation of harmful hydroxyl radicals. At the same time, it is also important to ensure enough iron for metabolic processes. The acid-induced protein Dps has in *C. jejuni* shown to be involved in superoxide and peroxide defence [41] and it is likely that the induction of Dps is a consequence of the iron released upon acid stress.

The induced 19 kDa protein (Cj1659) is a well-conserved periplasmic protein in *C. jejuni* and *Campylobacter coli* species [50] which previously was found to be Fur like (ferric uptake regulator) and iron regulated [20]. The p19 system is associated with an ABC iron transport system (*cj1659cj1663*) [46] and up-regulation of the 19 kDa protein therefore indicates a way to control the intracellular iron level during acid stress.

The thioredoxin system is composed of both TrxB and NADPH. In *E. coli,* TrxB interacts with unfolded and denatured proteins in a way comparable with molecular chaperones which are involved in proper folding of mis-folded proteins after stress [51]. A similar function of TrxB in *C. jejuni* might be possible as a part of the acid defence mechanisms. TrxB might mediate alkyl hydroperoxide reductase (AhpC) as is the case of *H. pylori*[37,52].

During the acid stress response, the enzyme MogA was induced, which to our knowledge has not been related to acid response before. However, an unpublished microarray study supported our result with acid exposure conditions comparable with our study (HCl exposure at pH 5.0 in strain NCTC 11168). After 10 minutes up-regulation *mogA* was measured, but only on the limit of the statistical threshold (Arnoud van Vliet, personal communications). MogA catalyzes the incorporation of molybdenum (Mo) into molybdopterin to form molybdenum cofactor (MoCo), a cofactor in molybdoenzymes [53]. Some molybdoenzymes in *E. coli* contain a modified form of MoCo. By transferring a GMP (guanosine monophosphate) to the terminal phosphate of MoCo, a molybdenum guanine dinucleotide (MGD) is formed. MGD is present in the enzymes formate dehydrogenase (FdhA) and nitrate reductase (NapA) in *E. coli*[54,55]. The periplasmic two-subunit complex, *C. jejuni* NAP, is considered as an electron acceptor [56] and the enzyme is encoded by *napAGHBLD*[13]. The NapA is a ~105 kDa catalytic subunit protein that binds the cofactor MGD. Basically, during electron transport at low O_2_, the molybdenum-containing enzyme nitrate reductase reduces NO_3_^-^ to NO_2_^-^ by consuming 2 H^+^. A transcriptional profile of *C. jejuni* NCTC 11168 after exposure to HCl stress resulted in a transiently or constantly up-regulated *napGHB* and *fdhA*[24], indicating that MogA most likely is part of an acid stress response.

The weak induction of SodB and AhpC indicate that the enzymatic oxidative stress defence play a role during acid stress. AhpC eliminates oxidative damaging compounds by converting alkyl hydroperoxides to the corresponding alcohol [37], and during this reaction a proton is consumed. SodB eliminates the damaging super oxides (O_2_^-^) [37,57], and in this reaction, protons are also consumed thereby preventing acidification of the cytoplasm. The elimination of O_2_^-^ and H^+^ by SodB generates H_2_O_2_, another stress factor. If free Fe^2+^ is present in the cell, the produced H_2_O_2_ can form hydroxyl radicals (·OH), which may directly damage DNA. This may explain the induced production of Dps that reversibly binds iron. The produced H_2_O_2_ can be removed by catalase (KatA) which converts H_2_O_2_ to H_2_O and O_2_[37,57]. In contrast to a transcriptional study where an up-regulation of *katA* gene was noticed after acid exposure [24], induction of KatA was not observed in this proteomic study. Since *C. jejuni* is sensitive towards oxygen and lacks numerous oxidative stress regulators such as SoxRS and OxyR [13], the cell might be in a constantly oxygen-alert state in order to remove reactive oxygen species and damaging components from acid stress.

No induction of heat shock proteins (Hsps) as chaperones or proteases were observed during acid stress in this study. A transcriptional study found an up-regulation of *clpB*, *dnaK*, *grpE*, *groEL/ES* and *htrA*[24]. One explanation could be the sensitivity of 2D-gel-electrophoresis for proteomic analysis as mentioned above and the detection limit due to molecular size and isoelectric point (pI) of the proteins. The Hsps, ClpP and GroES have molecular masses close to the maximum and minimum detection size, respectively, and HtrA has a pI of 9.6 which is outside the pI range of the system used here.

Acid exposure of *C. jejuni* NCTC 11168 was related to changes in gene expression and synthesis of acid stress proteins. However, comparison of the proteomic and transcription study showed a limited correlation between induced proteins and over-expression of genes. A recent proteomic study with acid adaptation of *Salmonella enterica* also [26] found a limited correlation between the outcomes of the transcriptional (qRT-PCR) versus translational (2D-gel) studies. The lack of corresponding results may be due to the lifetime of the RNA and the time from transcription to translation.

## Conclusions

It can be concluded that the three *C. jejuni* strains, at the phenotypic and proteomic level, responded differently to the acid stresses. We demonstrated that acid stress induces production of several proteins normally involved in iron control and oxidative stress defence in *C. jejuni*. This work has contributed to the understanding of what occurs in the *C. jejuni* cells during acid stress.

## Authors contributions

TIBIR: performed all experiments, analysed data, wrote the paper and calculated the statistics. MTW: involved in the qRT-PCR. RLA: Helped with the setup of 2D-gel electrophoresis, data analysis of 2D-gel experiments and correction of paper. SKN: supervising, discussion of results and revision of the manuscript. All the authors have given approval of the manuscript.
